# Interactions of Coated-Gold Engineered Nanoparticles with Aquatic Higher Plant *Salvinia minima* Baker

**DOI:** 10.3390/nano11123178

**Published:** 2021-11-24

**Authors:** Ntombikayise Mahaye, Melusi Thwala, Ndeke Musee

**Affiliations:** 1 Emerging Contaminants Ecological and Risk Assessment (ECERA) Research Group, Department of Chemical Engineering, University of Pretoria, Pretoria 0028, South Africa; mahaye.ntombi@gmail.com; 2 Water Centre, Council for Scientific and Industrial Research, Pretoria 0184, South Africa; mthwala@csir.co.za

**Keywords:** aquatic higher plants, *Salvinia minima* Baker, adsorption, accumulation, biomass, gold engineered nanoparticles

## Abstract

The study investigated the interactions of coated-gold engineered nanoparticles (nAu) with the aquatic higher plant *Salvinia minima* Baker in 2,7, and 14 d. Herein, the nAu concentration of 1000 µg/L was used; as in lower concentrations, analytical limitations persisted but >1000 µg/L were deemed too high and unlikely to be present in the environment. Exposure of *S. minima* to 1000 µg/L of citrate (cit)- and branched polyethyleneimine (BPEI)-coated nAu (5, 20, and 40 nm) in 10% Hoagland’s medium (10 HM) had marginal effect on biomass and growth rate irrespective of nAu size, coating type, or exposure duration. Further, results demonstrated that nAu were adsorbed on the plants’ roots irrespective of their size or coating variant; however, no evidence of internalization was apparent, and this was attributed to high agglomeration of nAu in 10 HM. Hence, adsorption was concluded as the basic mechanism of nAu accumulation by *S. minima*. Overall, the long-term exposure of *S. minima* to nAu did not inhibit plant biomass and growth rate but agglomerates on plant roots may block cell wall pores, and, in turn, alter uptake of essential macronutrients in plants, thus potentially affecting the overall ecological function.

## 1. Introduction

Gold engineered nanoparticles (nAu) find widespread use in cancer therapy [1,2], as nanocarriers in drug delivery [3], and as catalysts [4]. This is due to their unique optical properties, low inherent toxicity and relatively simple surface functionalization [5], and therefore, are released into the ecosystems [6,7]. However, despite a large body of published literature on the effects and transformations of nanoparticles (NPs) in ecosystems to date, there remain information gaps on their interactions with plants, especially at sub-lethal levels [8,9]. Studies on the interactions between aquatic higher plants and NPs have largely focused on silver (nAg), titanium dioxide (nTiO_2_) and zinc oxide (nZnO) as attested by recent reviews [8,10,11,12], with limited data on nAu. However, nAu has a relatively low dissolution rate compared to nZnO, nCuO and nAg [3,13,14]. Thus, it is more likely to accumulate in the environment. For example, nAu concentrations ranging from 0.13–0.25 μg/L have been detected in drinking water [15]. This, in turn, can potentially bioaccumulate and biomagnify in the food chain [16].

In the ecosystems, NPs can interact with aquatic higher plants—a class of plants among primary energy-producing organisms; hence, it can act as a potential reservoir, and source of NPs for subsequent transfer to higher trophic levels [8,17]. Interactions of NPs with aquatic higher plants have been reported as being influenced by numerous factors such as plant species [18,19], NPs’ size [20], NPs morphology [21], dissolution rate of the NPs [22,23], NPs surface properties [24,25,26], NPs exposure concentration [27,28], and environmental conditions such as the presence of UV radiation [29] but the findings were inconclusive and contradictory. For example, Glenn et al. [20] investigated the uptake of different-sized nAu (4 and 18 nm) at 250 µg/L to three morphologically distinct aquatic higher plants (*Myriophyllum simulans* Orchard., *Egeria densa* Planch., and *Azolla caroliniana* Willd.). Irrespective of size, nAu were internalized by *A. caroliniana*, only 4 nm-sized were internalized by *M. simulans*, and none by *E. densa*. Findings indicated that internalization was NPs size and plant species dependent. Conversely, no evidence of nAu (5 and 20 nm; 10 and 50 µg/mL) accumulation was observed in *Hordeum vulgare* L. Barley roots irrespective of their size and exposure concentration [30].

To date, there is limited data on the interactions of NPs with aquatic higher plants to draw firm conclusions, thus: (i) the influence of NPs physicochemical properties and exposure media chemistry are not well established, and/or (ii) where such linkages have been reported, exposure concentrations used are unrealistically high, relative to those in actual environmental matrices (e.g., freshwater, sediments, etc.) as previously reported from modeling [6,31], and experimental [15] studies. Consequently, published data based on high dosage are unlikely to support robust risk assessment of NPs in the aquatic systems. To address this knowledge gap, this study investigated interactions of the free-floating aquatic higher plant *Salvinia minima* Baker with nAu at 1000 µg/L, representing low but detectable concentration in the exposure media. Herein, preliminary studies showed that nAu concentrations <1000 µg/L (e.g., 62.5, 125, 250, and 500 µg/L) were below detection limits or could not be detected using Scanning and Transmission Electron Microscopy as well as Dynamic Light Scattering techniques. In addition, at these concentrations, no visible signs of toxicity (e.g., necrosis or growth retardation) were apparent on *S. minima*. Further, to increase the environmental realism of the study, NPs concentrations exceeding 1000 µg/L were not considered as they were deemed environmentally unrealistically high, hence, only 1000 µg/L concentration was used.

In this study, the specific objectives were to determine the influence of nAu physicochemical characteristics specifically their (i) size (5, 20, and 40 nm), and (ii) surface coating (citrate (cit) and branched polyethyleneimine (BPEI)) on their interactions with *S. minima*. The choice of *S. minima* as a model exposure plant is because it can easily be cultured under laboratory conditions, has a high growth rate, rapidly accumulates metals, and provide the necessary plant biomass for ecotoxicological assessments [32].

## 2. Materials and Methods

### 2.1. Characterization of nAu

Commercial cit- and BPEI-coated nAu suspensions were purchased from Nanocomposix (San Diego, CA, USA), and each type had three average sizes of 5, 20, and 40 nm according to the manufacturer specifications. The nAu were previously characterized for size and morphology elsewhere [33] using high-resolution transmission electron microscopy (HRTEM; JEOL JEM 2100, JEOL Ltd., Akishima, Tokyo, Japan) operating at 200 kV. Hydrodynamic diameter (HDD) and zeta (ζ) potentials of nAu in de-ionized water (DIW; 15 MΩ/cm), and 10% Hoagland’s medium (10 HM [34]; Sigma Aldrich, catalog number:H2395) were measured using dynamic light scattering (DLS; Malvern Zetasizer Nano ZS, Malvern Panalytical Ltd., Malvern, UK). The ζ potentials were calculated using Smoluchowski equation (Equation (S1)). In addition, aggregation of nAu in the exposure media was measured as the change in UV-Vis absorption spectrum using ultra-violet visible spectroscopy (UV-vis; HACH DR3900 spectrophotometer, Düsseldorf, Germany) at a wavelength range of 320 m–800 nm, using a quartz cuvette with a 1 cm optical path length. Measurements for ζ potential, HDD and UV-Vis spectra were taken at 0, 2, 6, 24 and 48 h, and in triplicates.

### 2.2. Preparation of Exposure Medium and Concentrations

The 10 HM (pH 7.1 ± 0.1; 65.64 mg/L Ca(NO_3_)_2_) was prepared by dissolving 0.16 g Hoagland-modified basal salt mixture purchased from Sigma Aldrich (Johannesburg, South Africa) in 1 L of DIW, and stored under dark conditions for 24 h before use. The composition of the media is shown in Table S1. The ionic strength (IS) of the exposure medium was calculated using the expression (Equation (S2)). Using exposure concentrations of 62.5, 125, 250, 500 and 1000 µg/L, electron microscopy findings indicated that only nAu at 1000 µg/L could be detected in *S. minima* tissues (roots and fronds). Hence, all studies were then conducted at 1000 µg/L. An exposure concentration of 1000 µg/L for each nAu type was prepared in 10 HM in triplicates, and bath sonicated for 30 min before initiating the experiments.

### 2.3. Test Organism Maintenance

Samples of *S. minima* were collected from Hartbeespoort Dam, North West Province, South Africa (25.7401° S, 27.8592° E), and transported in sampling site water to the laboratory. On arrival, plants were rinsed with tap water to remove attached debris, and thereafter, were acclimatized in a glass tank containing 5 L of 10 HM under natural light conditions at 21 ± 2 °C for 2 weeks. After every 5 d, culturing tanks were cleaned and the 10 HM solution was renewed.

### 2.4. Biomass Determination

Plants from the culturing tanks were dried on absorbent paper, and 500 mg fresh biomass of healthy plants was used as the test sample per replicate. Plants were exposed to 1000 µg/L of nAu in acid pre-washed glass beakers covered with transparent perforated parafilm to minimize evaporation. Roots were submerged in solution while the fronds floated; only the frond epidermis was in contact with the exposure media. Plants were then kept in a shaking incubator at 100 rpm for 14 d at 21 ± 2 °C under 16:8 h light: dark conditions, and 5000 lux light intensity. After 2, 7 and 14 d of exposure, whole plant samples were harvested and dried on absorbent paper for *ca* 1 min to remove water before determining the fresh biomass weight. Relative growth rate (RGR) was determined following expression [35].

(1) $$\mathrm{RGR}=\frac{lnW_{1}-lnW_{2}}{t}$$

where *W*_1_ and *W*_2_, respectively, represent the initial and final fresh weight (mg) and *t* as the incubation time (d).

### 2.5. Interactions of nAu with S. minima

#### 2.5.1. Determination of Total Au Concentrations

After 2, 7 and 14 d of exposure under the same exposure conditions described in Section 2.4, roots and fronds from nAu-exposed and non-exposed samples were separated, dried at 80 °C for 6 h in acid-washed and pre-weighed crucibles, followed by determination of dry weight after cooling. Next, the dried plant material in the crucibles was ashed at 530 °C for 12 h in a furnace (Delta DTA9696, Dewsbury, England). Using 250 μL HNO_3_, the ash was digested until fully dissolved, and the resultant solution was then diluted with DIW to achieve a 5% acid concentration. The resultant aqueous suspension was centrifuged at 3880× *g* for 10 min, and thereafter, total Au in the supernatant was measured using inductively coupled plasma mass spectrometry (ICP-MS) (Agilent ICP MS 7500cs, Agilent Technologies, Inc. 2006 Santa Clara, CA, USA).

#### 2.5.2. Internalization/Uptake of nAu by *S. minima*

After 14 d, plants were harvested, and roots and fronds were separated. Roots and fronds were fixed in 2.5% glutaraldehyde for 24 h and washed three times in 0.1M K-phosphate buffer pH 7.1 for 20 min, post-fixed with osmium tetroxide (OsO_4_) for 2 h, and rewashed by repeating the procedure. Plant samples were then dehydrated using increasing ethanol concentrations [35, 50, 70, and 90% (aqueous, *v*/*v*), and finally three times at 100%] for 20 min at each concentration, and left in 100% ethanol overnight. After dehydration, samples were then embedded into a 50:50 mixture of 100% ethanol: quetol epoxy resin for 1 h, followed by 100% quetol epoxy resin for 4 h, and finally into new 100% quetol epoxy resin and polymerized at 60 °C in a drying oven for 36 h before sectioning (90–100 nm thick) using an ultra-microtome. All samples were examined using TEM coupled with energy dispersive X-ray spectroscopy (EDX) to determine internalization of nAu by *S. minima*.

#### 2.5.3. Adsorption of nAu by *S. minima*

After 14 d exposure under the same conditions as described in Section 2.4, roots and fronds were separated, covered with aluminium foil and snap frozen in liquid nitrogen. The samples were stored at −80 °C until freeze-drying was conducted using Advantage Pro Lyophilizer (SP Scientific, Gardiner, NY, USA). Following freeze-drying, samples of roots and fronds were mounted on stubs, carbon coated then examined using scanning electron microscopy (SEM) [JOEL JSM 7500F, Japan with secondary electron (SE) detector at an acceleration voltage of 2 kV]; coupled with the EDX detector to map Au distribution at 15 kV.

### 2.6. Data Analysis

All data were presented as mean (*n* = 3) ± standard deviation (SD). The significance of comparisons between treatments was determined using one-way Analysis of Variance (ANOVA) at *p* < 0.05 using GraphPad Prism 7.04 Software (Graph Pad Prism software, La Jolla, CA, USA).

## 3. Results and Discussion

### 3.1. Characterization of nAu

**Figure 1:**
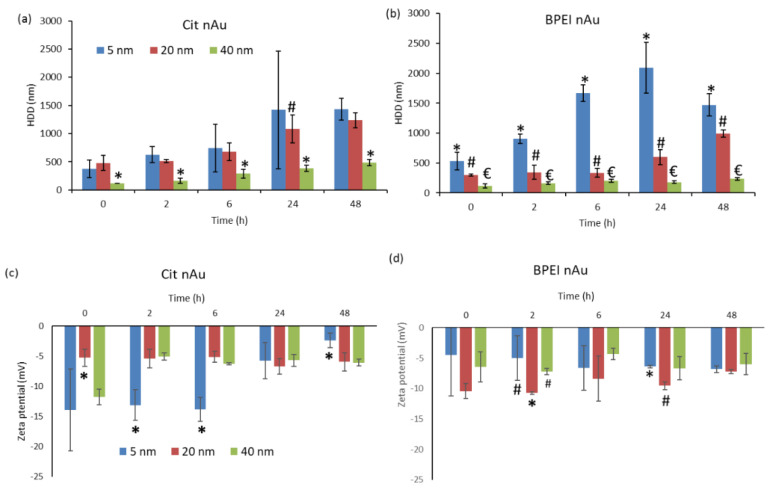
Hydrodynamic diameter (**a**,**b**) and zeta potentials (**c**,**d**) of gold nanoparticles at 1000 µg/L in 10% Hoagland’s medium measured using Dynamic Light Scattering technique over 48 h. Data are presented as mean (*n* = 3), bars denote standard deviations (SD), and different symbols denotes significant differences between nAu sizes per time period. nAu concentrations < 1000 µg/L were below detection limit using Zetasizer.

Cit- and BPEI-coated nAu had mixed morphologies consisting predominantly of spherical and a few rod and pentagon shapes (Figure S1). The measured mean sizes (*n* = 100) were similar to manufacturer’s specifications (Table S2). Here, “*n*” refers to the total number of individual nAu measured from TEM images to give the reported mean size. The particle size distributions of nAu in 10 HM at 0 h are shown in Figure S2. The 5 nm nAu rapidly agglomerated relative to larger counterparts (20 and 40 nm) in both DIW (Figure S3) and 10 HM (Figure 1a,b). This trend was consistent irrespective of coating type. The observed agglomeration was size-dependent owing to high surface energy, and large surface area for smaller sized-NPs [36,37,38]. Notably, the agglomerates size increased with increasing exposure time irrespective of their size and coating type (Figure 1a,b). Similarly, Mahaye et. al. [39] reported a similar trend following exposure of nAu (5, 20, and 40 nm; cit- and BPEI-coated; 1000 µg/L) to 10% BG-11 algal media for 72 h. Gold NPs had negative ζ potential in 10 HM (Figure 1c,d), and DIW (Figure S4)–with higher absolute value in the latter. The low ζ potential in 10 HM was due to the screening effect because of high ionic strength in the media [40]; indicative of nAu instability as evidenced by the observed rapid agglomeration. Although the manufacturer’s data indicated that BPEI-coated nAu had positive ζ potential (5 nm: not reported; 20 nm: +51.5 mV at pH 6.1; 40 nm: +49.1 mV at pH 7) in DIW, current findings showed that all sizes were negatively charged in both DIW (Figure S4) and 10 HM at pH 7 (Figure 1c,d). However, the cause for zeta potential alteration could not be established, although DLS experiments were repeated a number of times. The BPEI coating as it contains amine groups bears a cationic signature, hence, it ought to have remained positive. For example, in an inert medium such as the DI water one would not have expected any change; yet a negative charge was observed. Therefore, it is plausible that inaccuracies occurred in the reporting of the characterization data by the manufacturer. Hence, these results indicate the need to always confirm and characterize NPs’ physicochemical properties under study exposure conditions to aid draw firm conclusions.

**Figure 2:**
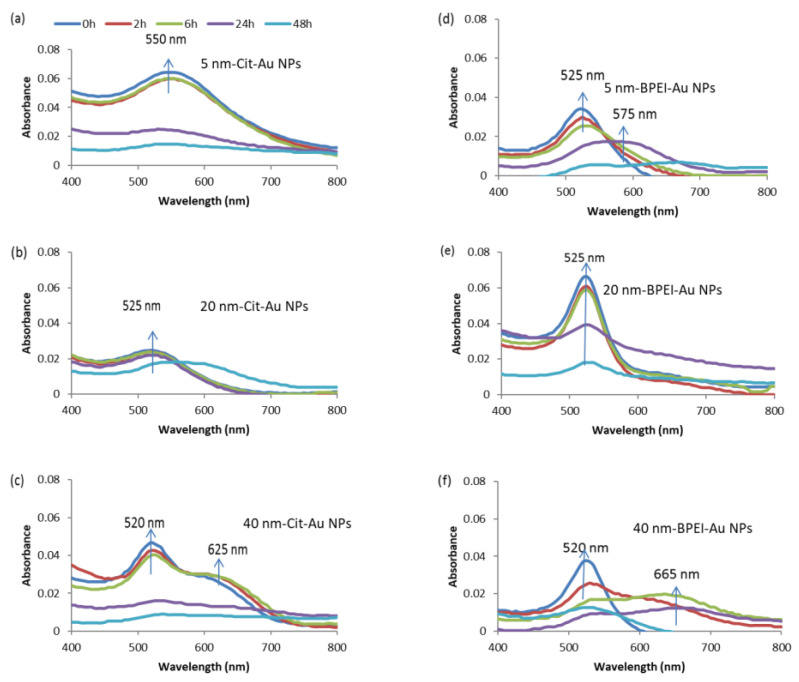
UV-vis spectra analysis of gold NPs in 10% Hoagland’s medium as a function of time, (**a**) 5 nm Cit-nAu, (**b**) 20 nm Cit-nAu, (**c**) 40 nm Cit-nAu, (**d**) 5 nm BPEI-nAu, (**e**) 20 nm BPEI-nAu, and (**f**) 40 nm BPEI-nAu. Data is presented as means (*n* = 3). Arrows show the position of the main peak.

Figure 2 depicts changes in the UV-Vis absorption spectra of nAu in 10 HM. The nAu were unstable in 10 HM (Figure 2) compared to DIW (Figure S5), as evidenced by either (i) a decrease in absorbance at λ_max_, (ii) peak shift with an appearance of a second peak at longer wavelengths (ca. 600 nm), and/or (iii) broadening of peaks. Such observations pointed to a loss of particle concentration (through sedimentation) from the solution due to agglomeration [41] driven by the high ionic strength of 10 HM compared to DIW. This is also evidenced by the decrease in particle concentration from the solution as NPs form agglomerates (Figure S6). The cit-coated nAu in 10 HM were stable over the first 6 h (Figure 2a–c); thereafter, peak broadening and decrease in absorbance were observed. The changes in UV-vis spectra were size-dependent (Figure 2a–c). The maximum absorption peaks decreased with increasing nAu size as follows: 5 nm cit-nAu (550 nm), 20 nm cit-nAu (525 nm), and 40 nm cit-nAu (520 nm). Previously, spherical-shaped cit-coated nAu (36 ± 7 nm) showed a maximum absorption peak at 521 nm in tap water [42]. Further, cit-coated nAu were unstable in biological buffers and artificial seawater, displaying significantly increased sizes, whereas no significant alterations were apparent for polyvinylpyrrolidone (PVP)-, polyethylene glycol (PEG)- and bovine serum albumin (BSA)-coated nAu attributed to formation of complexes between nAu and the coating [43]. For BPEI-nAu, the absorbance started to decrease after 2 h, and a second peak was observed at ca. 600 nm, attributed to increase in aggregate size.

In DIW, changes in UV-vis spectra were size-dependent since the maximum absorption peaks for 5, 20 and 40 cit-nAu were, respectively, at 510, 520 and 525 nm (Figure S4a–c). However, size-dependent influence was not observed for BPEI–nAu as all sizes peaked at 525 nm (Figure S4d–f). Earlier studies have reported that similar sized nAu with different coatings exhibited different stabilities [44], with BPEI-coated particles being more stable relative to cit-coated ones [45]. Furthermore, current findings are in good agreement with the results of Feichtmeier et al. [46] where the maximum absorption of 20 nm cit-nAu in hydrosol was observed at 523.5 nm. Overall, stability of nAu was dependent on their size, surface coating, and the properties of the exposure media, consistent with previous studies [47,48].

### 3.2. Fresh Biomass

**Figure 3:**
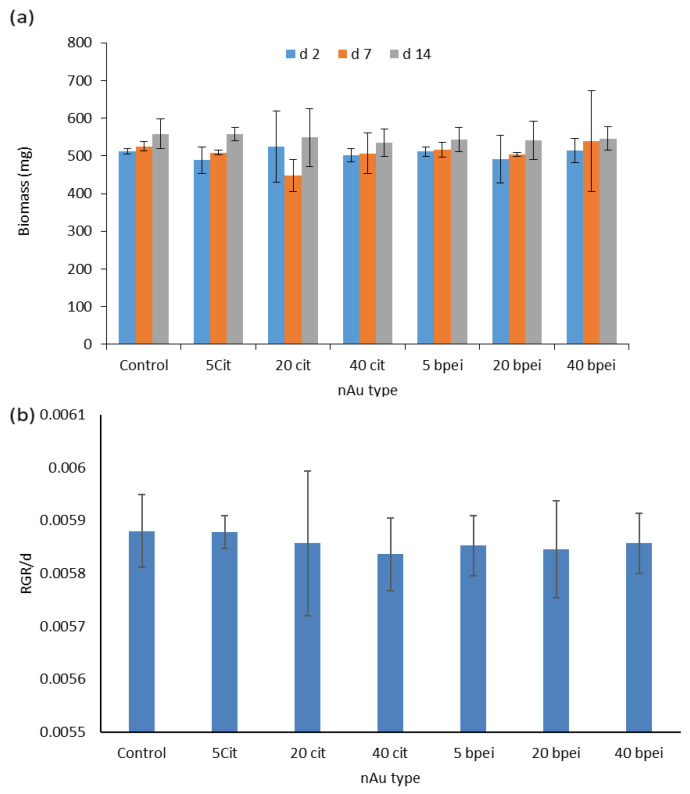
Plant growth for untreated and gold NPs-treated plants in 10% Hoagland’s medium over 14 d (**a**) fresh biomass (mg), (**b**) relative growth rate (RGR) per day. Results are reported as mean ± SD (*n* = 3), and bars denote standard deviations (SD). Using one-way ANOVA, no significant differences were observed between the controls and nAu-exposed samples over 14 d (*p* > 0.05), irrespective of endpoint.

Exposure of *S. minima* to 1000 µg/L nAu for 14 d did not significantly affect biomass and relative growth rate (RGR) (*p* > 0.05) compared to the controls irrespective of nAu size, coating type, and exposure duration (Figure 3a,b). The findings demonstrated that growth of *S. minima* was marginally affected by nAu under the present study test conditions. Similarly, nAu at 62 mg/L were reported to exhibit no inhibitory effects on plant growth and biomass attributed to activation of repair mechanism [49]. Findings confirmed the commonly known relatively low toxicity potential of nAu.

For instance, high exposure nAu concentrations (1.18–3.64 mg/L) did not induce toxic effects in *Ceratophyllum demersum* L but phytotoxicity was observed after 17 d [50]. Growth inhibition was observed at high concentration of 10 mg/L following exposure of *H. vulgare* to cit-coated nAu (10 nm, 1–10 mg/L) for 21 d; however, at a lower concentration of 1 mg/L, growth stimulation was observed [46]. Further, following exposure of *Salvinia auriculata* (Salvinaceae) to 1–10 mg/L nAg, an increase in biomass was observed at 1 mg/L, but a decrease occurred at 5 and 10 mg/L attributed to Ag interference with nutrient uptake [51]. Findings indicate that the biological effects of NPs in plants are dependent on exposure concentration, and duration. In addition, genotoxicity testing of nAu reference materials (10, 30 and 60 nm) at 0.2 µg/mL on HepG2 cells and calf-thymus DNA showed no evidence of DNA damage, and free radicals were not detected [52]. Notably, in previous, studies concentrations that induced biological effects were deemed too high and unlikely to be found in the ecosystems.

### 3.3. Total Au Analysis in Plant Tissues

**Figure 4:**
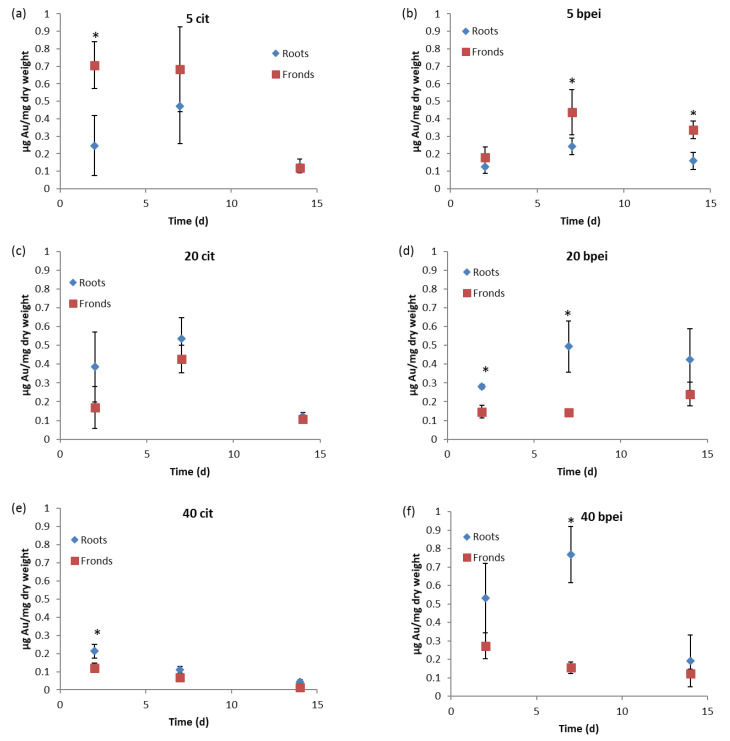
Gold concentrations (µg/mg dry weight) on *S. minima* exposed to 10% Hoagland’s medium at 1000 µg/L, (**a**) 5 nm Cit-nAu, (**b**) 20 nm Cit-nAu, (**c**) 40 nm Cit-nAu, (**d**) 5 nm BPEI-nAu, (**e**) 20 nm BPEI-nAu, and (**f**) 40 nm BPEI-nAu. Results are presented as mean (*n* = 3), bars denote standard deviations (SD), and * denotes significant differences between roots and fronds per time period using Two-way ANOVA at *p* < 0.05.

Spectrascan 100 mg/L Au 10% HCl *v/v* solution was used as quality control for the analysis of Au. The recovery (%) of Au by ICP-MS was 94.89–108.14%. Figure 4 depicts the concentrations of Au in plant roots and fronds analyzed using ICP-MS. Au concentrations in both roots and fronds for the control were below detection levels (<0.001 µg/mg). Concentrations on samples exposed to 5 nm-sized nAu (both coatings) were higher on the fronds than on the roots (Figure 4a,b). Herein, Au was observed to have accumulated both on the roots and fronds. These findings are similar to those of Das and Goswami [53] where Cu accumulation in *Salvinia cucullata* Bory. was observed on both roots and fronds. Findings for 20 and 40 nm-sized nAu showed that plant roots generally accumulated higher Au concentrations compared to fronds (Figure 4c–f); hence, results are in agreement with the literature [54,55,56]. For instance, Conway et al. [55] reported high concentrations of Ti and Ce in roots compared to fronds in *Clarkia unguiculata* Lindl. exposed to nTiO_2_ (194 ± 7 nm) and nCeO_2_ (231 ± 16 nm) at 1–100 mg/L for 8 w.

Aquatic plants are known to absorb nutrients through both roots and fronds [57]. The observed different trends on the adsorption of nAu based on size to *S. minima*, e.g., 5 nm nAu being higher on fronds but low on roots compared to larger forms is unclear. Although NPs can be translocated from roots to fronds [21,23,58], and whilst absorption of nutrients in aquatic higher plants can also occur via fronds, currently, there is no credible justification why the process was only selective for 5 nm-sized nAu.

High concentrations of Au except for 40 nm cit-nAu on both roots and fronds at day 7 relative to day 2, and a decrease to a minimum at day 14 suggests likely detachment of adsorbed NPs from the roots or fronds surfaces back into the solution. nAu adsorbed on plant roots can be released back to the solution as the exposure concentration decreases [46]. For both 40 nm-sized nAu, Au concentrations on roots decreased with increasing exposure period (Figure 4e,f). Concentration loss is linked to agglomeration facilitated by the sedimentation of NPs from suspension, which, in turn, lead to lower Au concentrations in suspension available for plants uptake.

### 3.4. Mechanism of nAu Accumulation by S. minima

**Figure 5:**
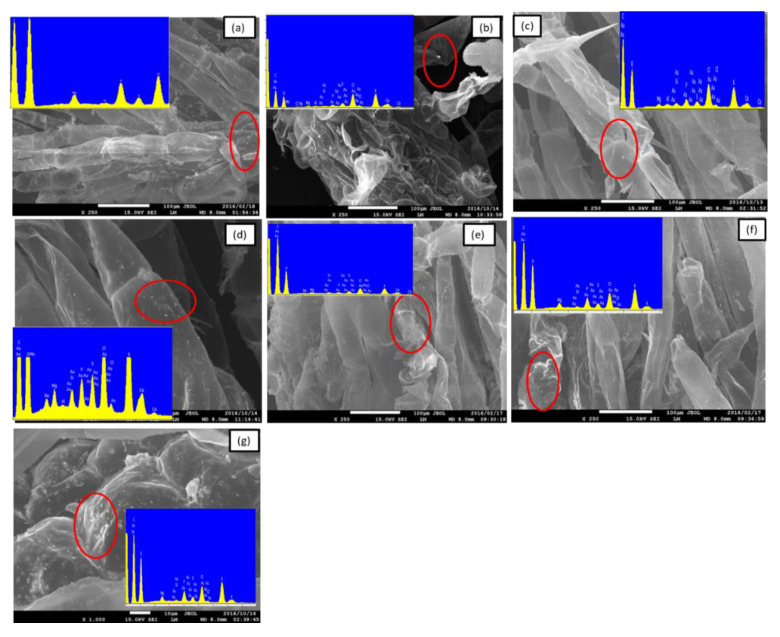
SEM images for nAu-treated and non-treated plant roots (**a**) control, (**b**) 5 nm-Cit, (**c**) 20 nm-Cit, (**d**) 40 nm-Cit, (**e**) 5 nm-BPEI, (**f**) 20 nm-BPEI, and (**g**) 40 nm-BPEI. Red circles indicate spots where EDX scan was taken, and the insert is the EDX spectra. Peaks indicate that gold, carbon, oxygen, magnesium, calcium, and silicon were all identified.

TEM and SEM were used to investigate the mechanism of nAu accumulation (internalization vs. adsorption) in *S. minima*. Results in Figure 5 demonstrated that interactions of *S. minima* with nAu occurred through roots surface adsorption. The presence of Au on roots surface was confirmed in all instances using EDX (insertions in Figure 5). TEM-EDX analysis was used to visualize the internalization of nAu in roots and fronds after 14 d. However, no evidence of nAu internalization into *S. minima* roots or fronds was observed irrespective of nAu size and coating variants (Figure S6). Thus, adsorption was concluded as the mechanism of nAu accumulation on *S. minima*. Similarly, 5 nm nAu (neutral, negatively, and positively charged) did not pass through the cell wall barrier of *Arabidopsis thaliana* L. (Arabidopsis) regardless of the surface charge, but assembled into clusters and were retained on the root surface [59]. Similar to our findings, Glenn et al. [20] reported adsorption of 18 nm nAu on *M. simulans* and *E. densa* roots without internalization into the cells. In addition, nAu (5 and 20 nm; 10 and 50 µg/mL) were not taken up by *H. vulgare* irrespective of size and exposure concentration [30]. Nanoparticle agglomerates were reported to be less likely internalized into cells and/or tissues, but may be adsorbed to cell membranes [59]. Other metal-based NPs (e.g., lead (nPb)) were adsorbed on *S. minima* cell walls in roots and fronds irrespective of their morphology (spherical or elongated) [60].

Numerous studies have reported accumulation of NPs in aquatic higher plants through either internalization- or adsorption-driven processes as summarized in Table 1. Generally, smaller-sized NPs are more rapidly internalized by aquatic higher plants [61]. However, data reveals that accumulation is dependent on complex and multifactorial factors, broadly categorized as physicochemical properties of NPs (e.g., size [20], coating type [62], morphology [60]), NPs type [63], exposure concentration [51,64], and plant physiological and phenotypic characteristics, e.g., presence of root hairs [20,65] (Table 1). Thus, in light of SEM and ICP-MS results (Figure 4 and Figure 5, respectively), adsorption mechanism accounted for the observed accumulation of nAu in *S. minima*. To date, only a handful of studies have reported internalization of NPs in aquatic higher plants [20,63,66,67] (Table 1).

In this study, the absence of internalization even for smaller-sized 5 nm nAu was attributed to their high agglomeration in 10 HM (Figure 1a,b). This is because the observed agglomerates (114–2095 nm) are larger than the cell wall pore size limit of ~10–50 nm [25,69]. For the same reason, among others, Taylor et al. [22] observed no internalization of nAu (5 and 100 nm) in *Medicago sativa* L. (alfalfa), but only ionic Au from AuCl_3_. In addition, plants were reported to respond to nAu exposure by up-regulating genes for plant stress and down-regulating specific metal transporters to reduce nAu uptake [22]. Due to the infancy of investigations on accumulation mechanisms of NPs in aquatic higher plants, these interactions remain poorly quantified, although they are key in elucidating likely implications to the ecological health. These data gaps can partly be attributed to lack of methodology necessary to determine the internalization and adsorption of NPs in higher aquatic plant tissues.

To date, studies on the interaction of *S. minima* with metal-based NPs are scarce; yet the plant is a hyperaccumulator of heavy metals such as manganese, lead and nickel [70,71,72]. Therefore, findings of the current study contribute to the limited body of knowledge on the interactions of metal-based NPs with *S. minima.*

## 4. Environmental Implications

Increasing production and widespread use of nAu have led to their release and accumulation in the environment [7,16]. This, in turn, has led to their concomitant interactions with aquatic organisms including aquatic higher plants—a primary producer and food source to organisms, e.g., crustaceans [73]. As a result, aquatic higher plants not only can act as reservoirs of NPs [20,61] but a source of NPs for subsequent transfer to higher trophic levels [8,73]. Herein, results demonstrate an insignificant effect of nAu on biomass and growth rate over time. This is evidence that nAu had no inhibitory effects on *S. minima* growth under test conditions studied here. Adsorption of nAu was observed on roots and fronds of an aquatic higher plants. However, internalization of nAu was not observed on both roots and fronds. Even in the absence of internalization, aquatic higher plants remain at risk as aggregation of NPs on root surfaces can cause physical-linked damages to roots [74]. This occurs by blocking cell wall pores and water transport capacity [75]. This, in turn, can reduce the concentrations of macronutrients (e.g., Ca, K, Mg, and S) on the leaves, thus affecting the chlorophyll content [76]. This work contributes to the few, yet growing number of, studies on the interactions of NPs with aquatic higher plants to fully account for short- and long-term implications that these interactions may pose to ecological health.

In addition, the study indicates aquatic higher plants are potential models for phytoremediation, as evidenced by nAu size- and exposure duration-dependent increase in accumulation (Figure 4). For example, maximum accumulation was observed on day 7 for 5 and 20 nm nAu of both coating types. Conversely, maximum accumulation was observed at day 2 for 40 nm-nAu and decreased with increased exposure duration. The study highlights the influence of NPs’ size and exposure duration on the likely environmental safety aquatic higher plants may offer. Thus, the ecological implications of NPs to aquatic higher plants cannot be generalized even for the same parent NPs.

## 5. Conclusions and Future Perspectives

Findings showed that nAu were unstable in 10 HM, as evidenced by larger HDD, low ζ potentials, and shift in peak spectra towards longer wavelengths. Exposure of *S. minima* to nAu at 1000 µg/L for 14 d did not significantly affect plant biomass and growth rate. High concentrations of 5 nm nAu accumulated on the fronds compared to roots of 20 and 40 nm nAu, but no evidence of internalization was established. Lack of internalization and insignificant effect on biomass and growth rate was attributed to the dynamic transformation of nAu, such as high agglomeration in 10 HM, which, in turn, may have hindered their uptake by plants. This implies the importance of physicochemical properties of NPs, and exposure media chemistry on their uptake and accumulation by aquatic higher plants. Adsorption of NPs on roots surface was confirmed in all instances irrespective of NPs size, and coating variant. Thus, adsorption was established as the mechanism of nAu accumulation on *S. minima.*

Overall, results indicated that nAu can be adsorbed on *S. minima* roots and fronds without internalization. Adsorption of NPs to plants’ roots can contribute to green phytoremediation and environmental safety, as aquatic plants can be used to remove nano-pollutants from the aquatic systems. Therefore, more research in this area using a wide range of plant types is highly encouraged. Furthermore, studies on the effects of NPs at different life cycle stages of plants are recommended. Even though nAu did not exert deleterious effects on *S. minima* at the morphological level (e.g., biomass growth inhibition); low concentrations of NPs as found in the environment are likely to exert sub-lethal effects. Hence, further studies at different endpoints at the molecular level (e.g., chromosomal abnormalities, DNA damage, genome template stability, etc.) may offer better insights into the likely toxicological outcomes of NPs adsorbed on the roots and/or fronds of aquatic higher plants. Due to the variety of NPs and aquatic higher plants, there is a need for further research on how processes of adsorption and internalization occur under different complex and multifunctional scenarios, especially at environmentally realistic NPs concentrations, and in actual matrices, e.g., freshwater systems as opposed to synthetic media.

## Figures and Tables

**Table 1:** Mechanisms of NPs accumulation by aquatic higher plants.

Plant	Mechanism	Detection Method	ENP Type	ENP Properties	Exposure Media	Duration	Dosage	Controlling Factor	Ref.
*Azolla caroliniana*	Internalization	TEM, STEM, SEM, EDX	Au	4 nm; 18 nm; spherical −14.1 mV; ζ −9.73 mV	Borehole water; pH 7.1; TOC;8.56 mg/L; CaCO_3_ 107 mg/conductivity 210 mS/cm	24 h	250 µg/L	Species type: internalization due to the presence of root hairs used by the plant to acquire nutrients	[20]
*Egeria densa*	Adsorption	TEM, STEM, SEM, EDX	Au	4 nm; 18 nm; spherical; ζ −14.1 mV; ζ −9.73 mV	Borehole water; pH 7.1; TOC;8.56 mg/L; CaCO_3_ 107 mg/conductivity 210 mS/cm	24 h	250 µg/L	Presence of root hairs facilitated internalization	[20]
*Lemna minor*	Adsorption (cell wall of leaves)	SEM; TEM	TiO_2_	275–2398 nm; SSA 50 m^2^/g;	Steinburg growth medium, pH 5.5; CaCO_3_ 166 mg/L	14 d	0.01–10 mg/L	Exposure concentration: Accumulation increased with an increase exposure concentration	[64]
*Myriophyllum simulans*	Adsorption	TEM, STEM, SEM, EDX	Au	4 nm; spherical; ζ −14.1 mV	Borehole water; pH 7.1; TOC;8.56 mg/L; CaCO_3_ 107 mg/conductivity 210 mS/cm	24 h	250 µg/L	Size: High accumulation from 4 nm Au NPs.	[20]
*Salvinia auriculata*	Absorption	ICP-MS	Ag	100 nm, PVP-coated	Cultivation media with 14/10 h (light/dark) cycle and temperature between 23 and 24 °C in a greenhouse	64 d	1–10 mg/L	Absorption increased with exposure time, and nAg concentration	[51]
*Salvinia minima*	Adsorption (cell wall of leaves)	TEM, SEM, XPS	Pb	spherical, 17.2 ± 4.2 nm	Hoagland’s medium	12 h	80 mg/L	Morphology: Spherical NPs were found within the cell wall while elongated ones were associated with the cell membrane.	[60]
*Salvinia minima*	Adsorption (cell wall of roots)	TEM, SEM, XPS	Pb	Elongated, 53.7 ± 29.6 nm in length and 11.1 ± 2.4 nm wide	Hoagland’s medium	12 h	80 mg/L	Spherical shaped NPs were within the cell wall while elongated ones were associated with the cell membrane	[60]
*Salvinia minima*	Adsorption (roots and leaves)	TEM, SEM, ICP-MS	Au	5, 20, 40 nm; spherical; citrate and BPEI coated	10% Hoagland’s medium; pH 7	14 d	1 mg/L	Exposure media: high agglomeration of NPs leading to lack of internalization	[current study]
*Salvinia natans*	Adsorption	ICP-OES	ZnO	25 nm; uncoated; SSA; 90 m^2^/g; 1–10 mg/L	OECD growth medium; pH 6.5	7 d	1–50 mg/L	Concentration: High agglomeration and settling of NPs at 20 and 50 mg/L	[68]
*Schoenoplectus tabernaemontani*	Internalization (roots)	TEM	CuO; CdS QDs	38 nm; SSA 12.84 m^2^/g; ζ −2.8 mV	Hoagland’s medium	21 d	0.5–50 mg/L	NP type: Root uptake percentage for nCuO treatment ranged from 40.6 to 68.4%, while the values were 8.7 to 21.3% for CdS QDs	[63]
*Schoenoplectus tabernaemontani*	Internalization	SEM; TEM	ZnO	35 nm; SSA 43 m^2^/g; ζ −5.4 mV (start), −2.6 mV (end)	Nutrient solution, pH 6.4–6.8	21 d	10–1000 mg/L	Particulate vs. ionic form: Uptake of Zn from nZnO was greater than that for Zn^2+.^	[66]
*Spirodela polyrrhiza*	Internalization	Epifluorescence microscopy	TiO_2_	8 nm, anatase	50% *S. polyrrhiza* specific culture medium	6 d	0.05–10 mg/L	Structural characteristics: Anatase and crystalline nTiO_2_ allow their remarkable movement into the root cells	[67]

## Supplementary Materials

The following are available online at https://www.mdpi.com/article/10.3390/nano11123178/s1, Equation (S1): Calculation of ζ potentials using Smoluchowski equation, Equation (S2): Calculation of ionic strength (IS) of the exposure medium, Figure S1: TEM images of nAu (a) 5 nm-Cit, (b) 20 nm-Cit, (c) 40 nm-Cit, (d) 5 nm-BPEI, (e) 20 nm-BPEI, (f) and 40 nm-BPEI, Table S1: Composition of Hoagland’s medium, Table S2: Mean sizes (nm) of nAu obtained using TEM, Figure S2: Particle size distribution of nAu at 1000 µg/L in 10% Hoagland’s medium measured using Dynamic Light Scattering technique (a) 5 nm Cit-nAu, (b) 20 nm Cit-nAu, (c) 40 nm Cit-nAu, (d) 5 nm BPEI-nAu, (e) 20 nm BPEI-nAu, and (f) 40 nm BPEI-nAu, Figure S3: Hydrodynamic diameters of nAu in de-ionized water and 10% Hoagland’s medium tracked using Dynamic Light Scattering technique over 48 h; (a) 5 nm Cit-nAu, (b) 20 nm Cit-nAu, (c) 40 nm Cit-nAu, (d) 5 nm BPEI-nAu, (e) 20 nm BPEI-nAu, and (f) 40 nm BPEI-nAu, Figure S4: Zeta potentials of nAu in de-ionized water and 10% Hoagland’s medium obtained using Dynamic Light Scattering technique over 48 h; (a) 5 nm Cit-nAu, (b) 20 nm Cit-nAu, (c) 40 nm Cit-nAu, (d) 5 nm BPEI-nAu, (e) 20 nm BPEI-nAu, and (f) 40 nm BPEI-nAu, Figure S5: UV-vis spectrum of nAu in de-ionized water as a function of time; (a) 5 nm Cit-nAu, (b) 20 nm Cit-nAu, (c) 40 nm Cit-nAu, (d) 5 nm BPEI-nAu, (e) 20 nm BPEI-nAu, and (f) 40 nm BPEI-nAu, Figure S6: in situ nAu concentration (particles/mL) examined using Nanoparticle Tracking Analysis (NTA), Figure S7: TEM-EDX spectra confirming the absence of nAu internalization on plant roots: (a) control, (b) 5 nm cit-nAu, (c) 20 nm-cit nAu, (d) 40 nm cit-nAu, (e) 5 nm BPEI-nAu, (f) 20 nm BPEI-nAu, and (g) 40 nm BPEI.
