# Supplementary material for: Interactions of Coated-Gold Engineered Nanoparticles with Aquatic Higher Plant Salvinia minima Baker
Source: Nanomaterials (Basel). 2021 Nov 24;11(12):3178. doi: 10.3390/nano11123178 (PMC8704737; doi:10.3390/nano11123178)
Supplement: Supplementary file 1 [file nanomaterials-11-03178-s001.zip › nanomaterials-1413085-supplementary.pdf]

Supplemetary materials

Interactions of Coated-Gold Engineered Nanoparticles with Aquatic Higher Plant *Salvinia minima* Baker

Ntombikayise Mahaye [1], Melusi Thwala [2] and Ndeke Musee [1,*]

[1] Emerging Contaminants Ecological and Risk Assessment (ECERA) Research Group, Department of Chemical Engineering, University of Pretoria, Pretoria, 0028, South Africa; mahaye.ntombi@gmail.com

[2] Water Centre, Council for Scientific and Industrial Research, Pretoria, 0184, South Africa; mthwala@csir.co.za

* Correspondence: ndeke.musee@up.ac.za or museen2012@gmail.com

The ζ potentials were calculated using Smoluchowski equation:

$$\upsilon E = 4\pi\varepsilon 0\varepsilon r + \frac{\zeta}{6\pi\mu} + (1 + \kappa r) \qquad \text{(Eq. S1)}$$

where ε0 and εr are the relative dielectric constant and the electrical permittivity of a vacuum respectively, μ is the solution viscosity, r is the particle radius and $\kappa = (2n0z^2e^2/\varepsilon r\varepsilon 0kBT)^{1/2}$ is the Debye–Hückel parameter, *n0* is the bulk ionic concentration, *z* is the valence of the ion, e is the charge of an electron, kB is the Boltzmann constant, and T is the absolute temperature.

The ionic strength (IS) of the medium was calculated as follows:

$$\text{IS} = \frac{1}{2}\sum_i C_i Z_i^2 \qquad \text{(Eq. S2)}$$

where IS is the ionic strength in mM, C_i is the concentration of the ith species in mM, and Z_i is the charge of the ith species.

Table S1: Composition of Hoagland's medium

Macronutrient	Per litre of nutrient solution
KNO_3	5 ml of 1 M
$Ca(NO_3)_2$ $4H_2O$	5 ml of 1 M
$MgSO_4{\cdot}7(H_2O)$	2 ml of 1 M
KH_2PO_4	2 ml of 1 M
**Micronutrients**	**Per litre (g)**
H_3BO_3	2.86
$MnCl_2{\cdot}4(H_2O)$	1.81
$ZnSO_4{\cdot}7H_2O$	0.22
$CuSO_4{\cdot}5H_2O$	0.08
MoO_3	0.02
Fe-EDTA	1-5 ml of 1000 mg/L
**Minus nitrogen**	**Per litre of nutrient solution**
$Ca(NO_3)_2$ $4H_2O$	0.75 ml of 1 M
$Ca(H_2PO_4)_2{\cdot}H_2O$	10 ml of 0.05 M
$CaSO_4{\cdot}2H2O$	200 ml of 0.01 M
K_2SO_4	5 ml of 0.5 M
$MgSO_4.7H_2O$	2 ml of 1 M

Source: Hoagland and Arnon [1]

Table S2: Mean sizes (nm) of nAu obtained using TEM

Sample	Size (nm) (Manufacturer)	Size (nm) (Current study)
5 nm cit-nAu	4.8±0.7	5.6±1.4
20 nm cit-nAu	20.0±2.5	20.7±2.5
40 nm cit-nAu	39.9±4.1	41.3±4.0
5 nm BPEI-nAu	4.3±0.6	5.0±0.6
20 nm BPEI-nAu	18.2±1.6	19.0±2.7
40 nm BPEI-nAu	41.3±4.2	41.3±2.8

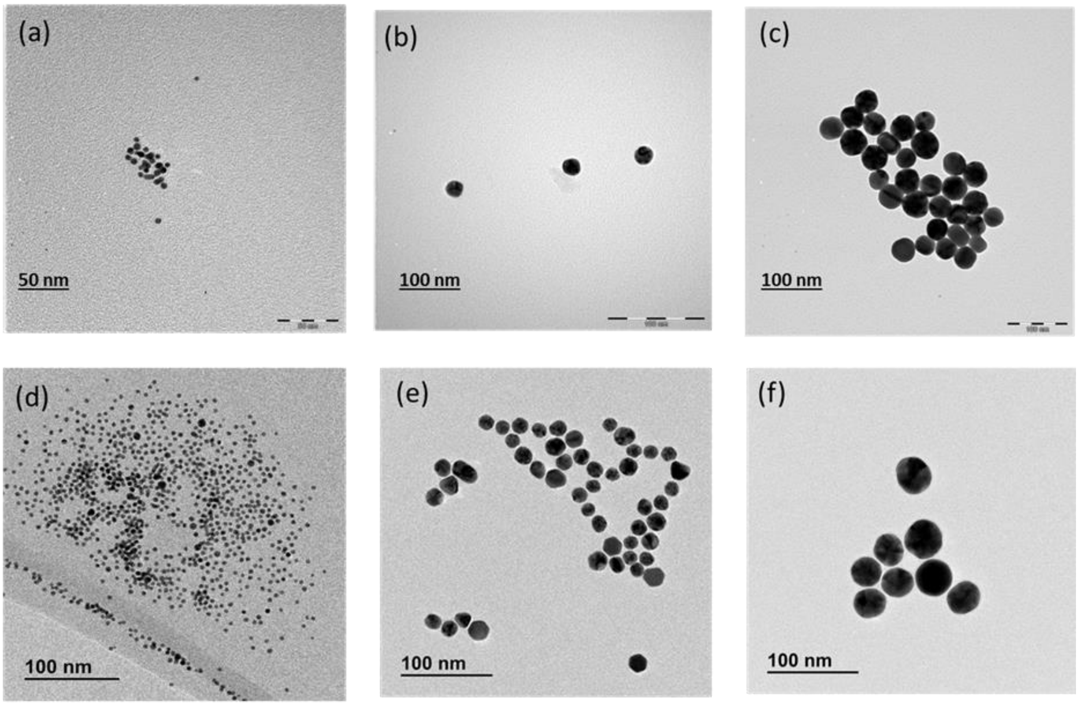

Figure S1. TEM images of nAu (**a**) 5 nm-Cit, (**b**) 20 nm-Cit, (**c**) 40 nm-Cit, (**d**) 5 nm-BPEI, (**e**) 20 nm-BPEI, (**f**) and 40 nm-BPEI. Source: Mahaye [2].

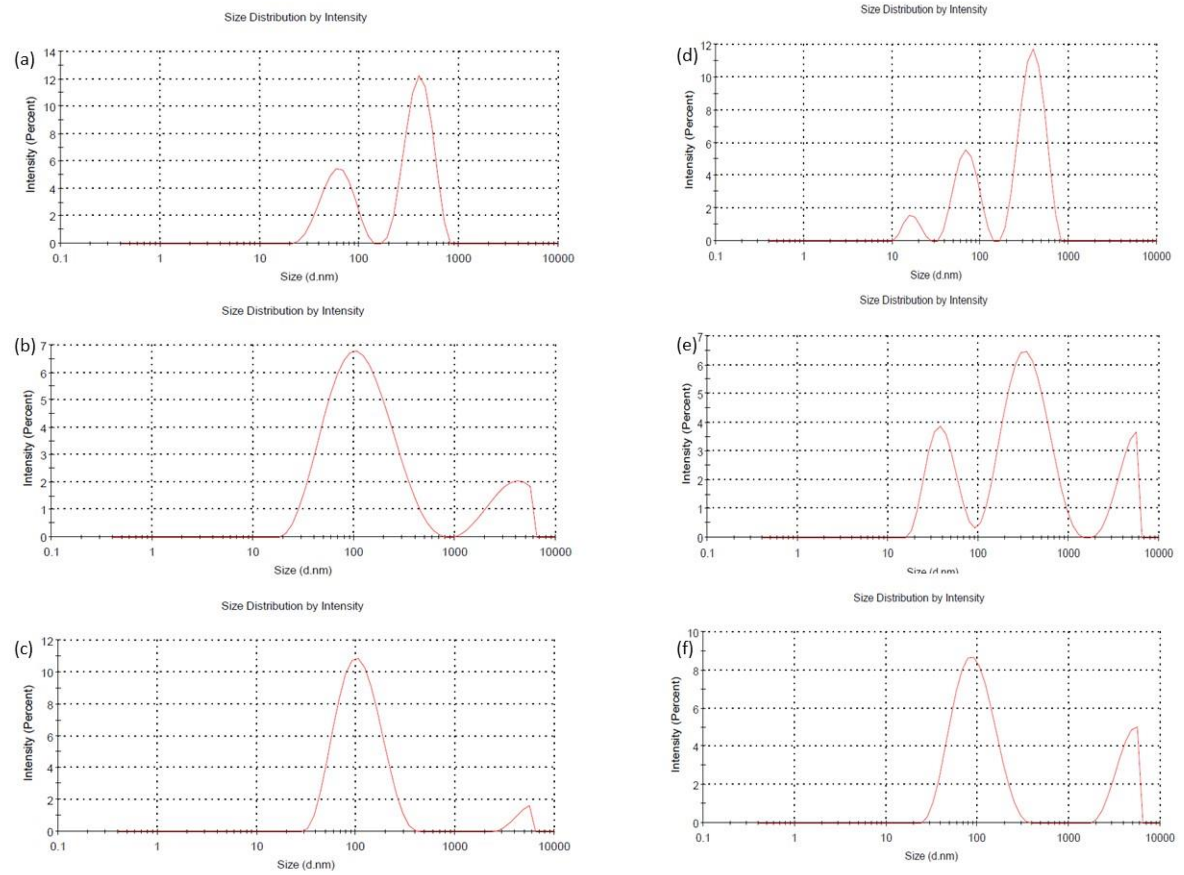

Figure S2: Particle size distribution of gold nanoparticles at 1000 µg/L in 10% Hoagland’s medium measured using Dynamic Light Scattering technique at 0 h (**a**) 5 nm Cit-nAu, (**b**) 20 nm Cit-nAu, (**c**) 40 nm Cit-nAu, (**d**) 5 nm BPEI-nAu, (**e**) 20 nm

BPEI-nAu, and (**f**) 40 nm BPEI-nAu.

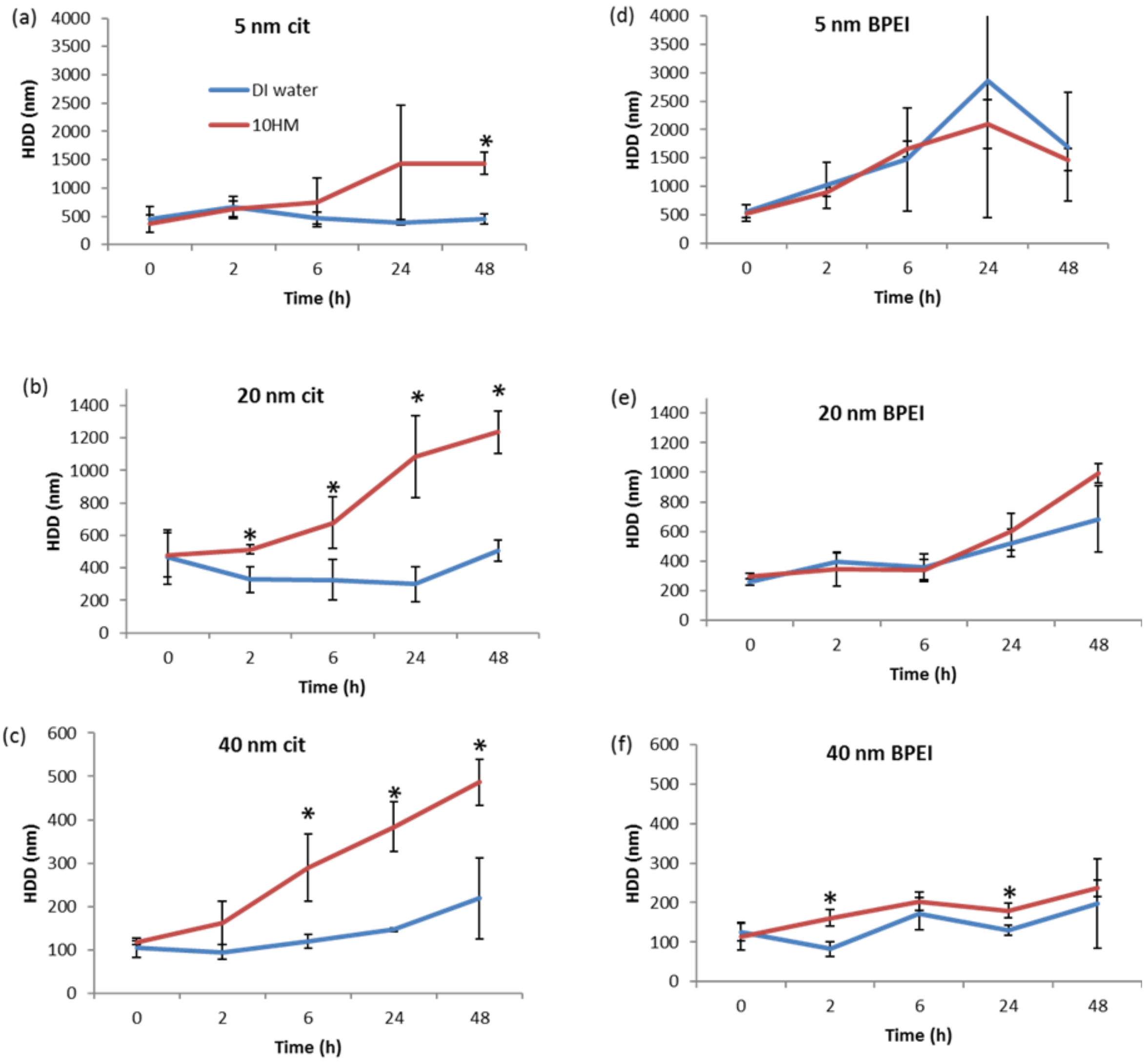

Figure S3: Hydrodynamic diameters of nAu in de-ionized water and 10% Hoagland's medium tracked using Dynamic Light Scattering technique over 48 h. (**a**) 5 nm Cit-nAu, (**b**) 20 nm Cit-nAu, (**c**) 40 nm Cit-nAu, (**d**) 5 nm BPEI-nAu, (**e**) 20 nm BPEI-nAu, and (**f**) 40 nm BPEI-nAu. Bars denote standard deviations, and statistical difference between the two media per time period is denoted by * where n = 3.

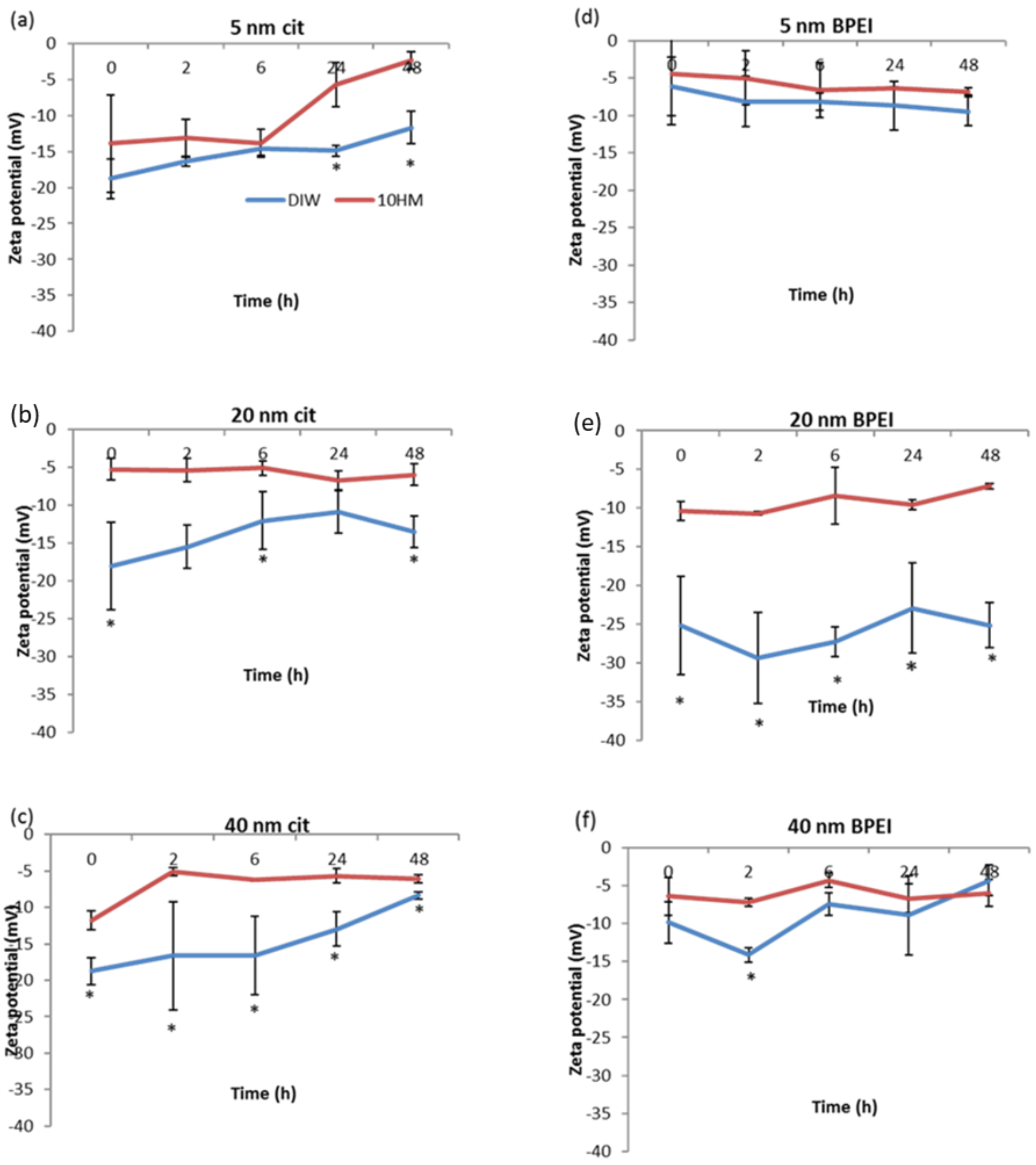

Figure S4: Zeta potentials of nAu in de-ionized water and 10% Hoagland's medium obtained using Dynamic Light Scattering technique over 48 h. (**a**) 5 nm Cit-nAu, (**b**) 20 nm Cit-nAu, (**c**) 40 nm Cit-nAu, (**d**) 5 nm BPEI-nAu, (**e**) 20 nm BPEI-nAu, and (**f**) 40 nm BPEI-nAu. Bars denote standard deviations, and statistical difference between the two media per time period is denoted by * where $n = 3$.

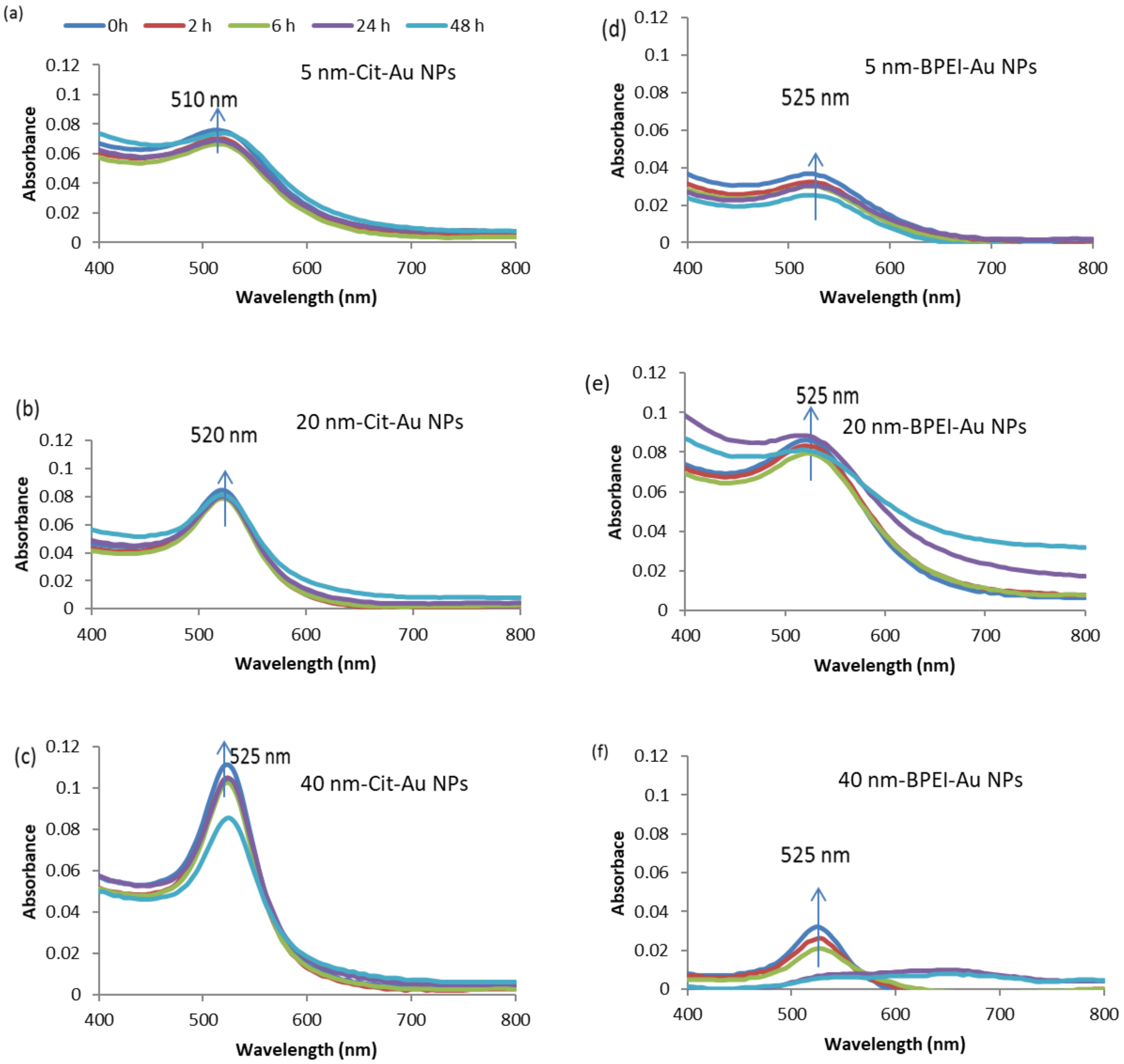

Figure S5: UV-vis spectrum of nAu in de-ionized water as a function of time. (**a**) 5 nm Cit-nAu, (**b**) 20 nm Cit-nAu, (**c**) 40 nm Cit-nAu, (**d**) 5 nm BPEI-nAu, (**e**) 20 nm BPEI-nAu, and (**f**) 40 nm BPEI-nAu. Data are presented as means (n = 3). Arrows show the position of the main peak. Source: Mahaye [2].

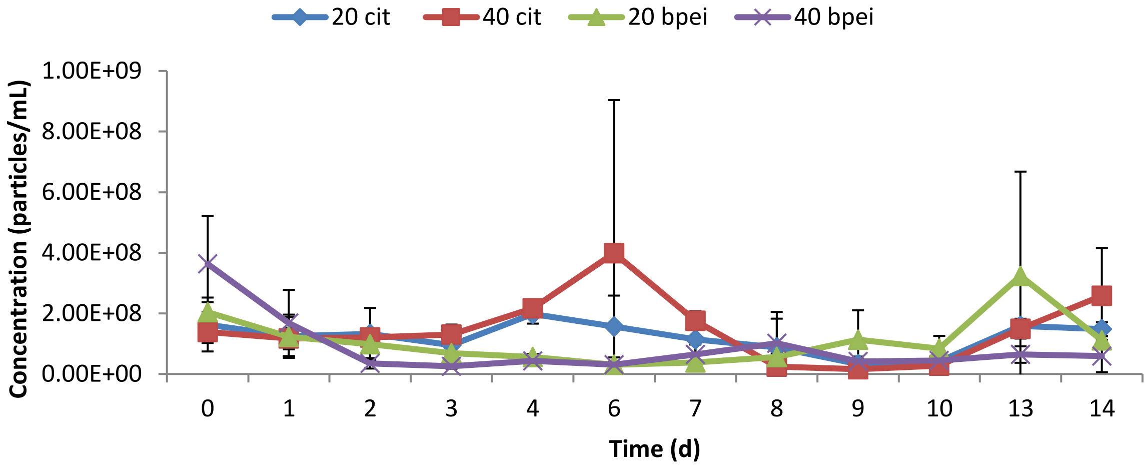

Figure S6: *in situ* nAu concentration (particles/mL) examined using Nanoparticle Tracking Analysis (NanoSight NS500, NTA 3.0 Software, Amesbury, UK). Results are represented as mean (*n* =3) and bars denote standard deviation. The 5 nm sized-nAu samples were excluded from NTA analysis as the technique cannot measure sizes below 10 nm accurately (NanoSight NS500 operating manual, Version P553S).

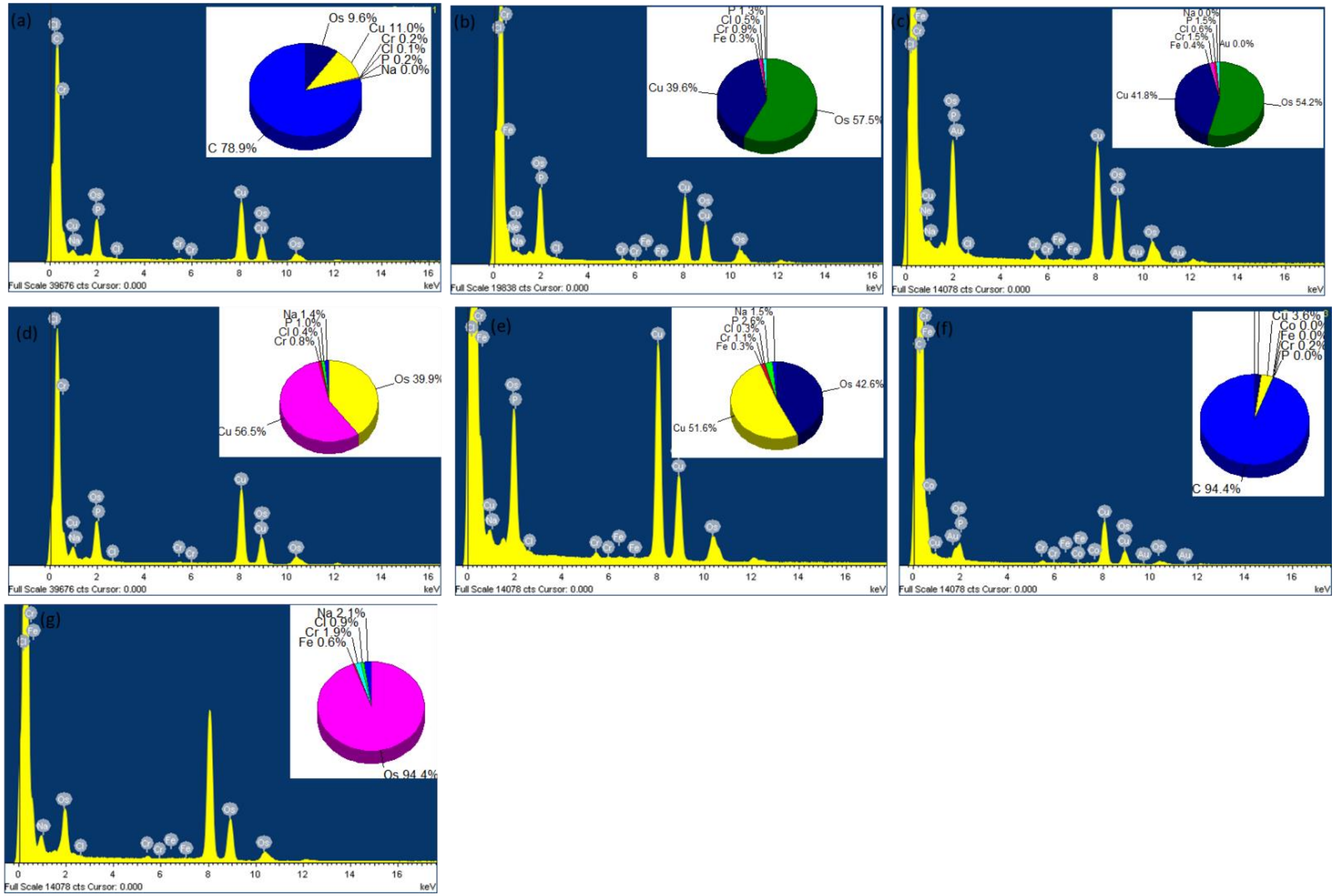

Figure S7: TEM-EDX spectra confirming the absence of nAu internalization on plant roots: (**a**) control, (**b**) 5 nm cit-nAu, (**c**) 20 nm-cit nAu, (**d**) 40 nm cit-nAu, (**e**) 5 nm BPEI-nAu, (**f**) 20 nm BPEI-nAu, and (**g**) 40 nm BPEI. Peaks indicate that only iron, cupper, sodium, osmium, chromium, chlorine, and phosphorus were identified. The inserts on Figures indicate percentages of the detected elements.

References

1. Hoagland DR; Arnon DI. The Water-Culture Method for Growing Plants without Soil. *California Agricultural Experiment Station Circular* **1950**, *347*, 1–32.
2. Mahaye, N. Stability of Gold and Cerium Oxide Nanoparticles in Aqueous Environments, and Their Effects on Pseudokirchneriella Subcapitata and Salvinia Minima. PhD, University of Pretoria: Pretoria, **2019**. https://repository.up.ac.za/handle/2263/72778.
